# Isolation and characterization of new antagonistic bacteria P10-7 and evaluation of its biocontrol potential against tomato gray mold

**DOI:** 10.3389/fmicb.2025.1668865

**Published:** 2025-09-18

**Authors:** Denghui Chen, Dandan Yue, Guanjie Li, Zongyuan Zhang, Liuzhu Zhou, Hongguang Xu, Dehai Liu, Xueyan Wang

**Affiliations:** ^1^Institute of Biology, Henan Academy of Science, Zhengzhou, China; ^2^Institute of Chemistry, Henan Academy of Science, Zhengzhou, China

**Keywords:** tomato gray mold, *Botrytis cinerea*, biocontrol strains, *Bacillus amyloliquefaciens*, inhibitory activity, stability, secondary metabolite, whole-genome

## Abstract

Gray mold, caused by *Botrytis cinerea*, is one of the most destructive fungal diseases, causing significant losses in cash crops, especially tomatoes, worldwide. To address this challenge, we isolated and characterized a novel bacterial strain, P10-7, from tomato rhizosphere soil. Identification was performed using morphological, physiological, biochemical, and whole-genome sequencing analyses. The biocontrol potential of P10-7 was assessed through *in vitro* antagonism assays, analysis of secondary metabolites and lytic enzymes, and greenhouse pot experiments. Results showed that P10-7 was a strain of *Bacillus amyloliquefaciens,* with a total genome size of 3,929,792 bp, including 12 biosynthetic gene clusters. The antagonism test demonstrated broad-spectrum antifungal activity against seven fungal pathogens, including gray mold, with inhibition rates of 92.09% for mycelial growth and 98.03% for spore germination. Biochemical tests confirmed the strain’s ability to produce amylase, protease, pectinase, and siderophores. Furthermore, application of P10-7 cell suspension at 1.0 × 10^7^ CFU/mL significantly promoted tomato seed germination and enhanced seedling growth (height, root length, fresh and dry weight). Critically, this treatment also markedly reduced disease incidence and effectively controlled tomato gray mold in greenhouse trials. Collectively, our findings demonstrate that *Bacillus amyloliquefaciens* P10-7 exhibits significant potential as an effective biocontrol agent against *Botrytis cinerea*.

## 1 Introduction

The tomato (*Solanum lycopersicum* L.) is an excellent model organism and one of the most widely consumed vegetable crops worldwide. Morever, gray mold, caused by *Botrytis cinerea*, is a polyphagous fungal pathogen capable of infecting more than 1,400 plant species and is the most serious postharvest fungal disease in tomatoes (Chen et al., 2023). It is estimated that gray mold causes global losses of fruit and vegetables, ranging from $10 billion to $100 billion annually. Chemical, physical and biocontrol methods have been applied to control *B. cinerea* (Bu et al., 2021). However, chemical fungicides cause serious environmental pollution, introduce harmful elements, lead to the development of resistant strains, pose risks to human health due to residues and hinder sustainable agricultural development (Yoon et al., 2024). Therefore, biocontrol methods based on microorganisms exhibit excellent potential for controlling plant pathogens due to their safety, eco-friendliness and high efficiency (Dengbo et al., 2022).

Microbial biological control agents (MBCAs) have emerged as viable alternatives to chemical fungicides and are recognized as key players in modern sustainable agriculture (Rebouh et al., 2022). To date, numerous fungi and bacteria exhibiting good biocontrol activity against plant pathogens, including gray mold, have been isolated from the rhizosphere of cultivated crops and identified, such as *Trichoderma harzianum* (Elad, 2000), *T. viride*, *Ulocladium* spp. (Köhl and Molhoek, 2008), *Clonostachys rosea* (Gong et al., 2017), *Gliocladium catenulatum*, *Saccharomyces cerevisiae*, *Wickerhamomyces anomalus*, *Metschnikowia pulcherrima* and *Aureobasidium pullulans* (Parafati et al., 2015; El-Saadony et al., 2022). Among them, *Bacillus* spp. play prominent role in inhibiting the growth and development of plant pathogens due to their wide distribution, extensive adaptability, genetic biodiversity and diverse biocontrol mechanisms (Ye et al., 2020; Wang et al., 2021). The *Bacillus* genus includes more than 228 species (Sumpavapol et al., 2009). However, only a few, including *B. subtilis* (Bu et al., 2021), *B. cabrialesii* (Guo et al., 2023), *B. tequilensis* (Jia et al., 2023), *B. altitudinis* (Zheng et al., 2023), *B. amyloliquefaciens* (Wang et al., 2024), *B. mojavensis* (Li et al., 2022) and *B. velezensi* (Li et al., 2022), have proven effective against tomato gray mold.

Metabolites produced by biocontrol strains are important for inhibiting the growth of the pathogen and are highly stable (Li et al., 2020; Le Han et al., 2022). Therefore, it is necessary to explore and isolate more possible evolutionary *Bacillus* strains in time to control *B. cinerea*.

Therefore, a bacterial strain (P10-7) exhibiting antagonistic activity against *B. cinerea* was isolated from tomato rhizosphere soil. This strain was characterized using morphological, physiological, biochemical, and whole-genome sequencing approaches. We further investigated the antagonistic effects of P10-7 cell suspension and its sterile fermentation filtrate on the pathogen, analyzed the stability of its antimicrobial metabolites, and evaluated its plant growth-promoting activity and biocontrol efficacy against gray mold in tomato seedlings through pot experiments. Additionally, the biocontrol mechanisms were explored through genomic analysis. This work provides a theoretical foundation for the biological control of tomato gray mold.

## 2 Materials and methods

### 2.1 Tested strains and culture conditions

A total of 40 soil samples were collected from the rhizosphere of various crops (tomato, cucumber, cabbage, pepper, watermelon; 8 samples per crop). From these, *B. amyloliquefaciens* P10-7 was isolated from tomato rhizosphere soil and deposited in the China General Microbiological Culture Collection Center (CGMCC) as a patent strain. In addition, *B. cinerea* was obtained from CGMCC and cultured on potato dextrose agar (PDA) medium at 25 °C. Information on the three bacterial and seven fungal pathogens used is listed in Table 1. PDA medium (20 g glucose, 200 g potato, 15–20 g agar, 1,000 mL distilled water, pH 7.0) and LB medium (5 g yeast extract, 10 g peptone, 10 g NaCl, 15–20 g agar, 1,000 mL distilled water, pH 7.0) were used.

**Table 1:** The bacterial and fungal strains tested in this study.

Strain	Characteristics relevant to this work	Source
1	*Pythium aphanidermatum*	This lab
2	*Didymella glomerata*	This lab
3	*Colletotrichum scovillei*	This lab
4	*Fusarium oxysporum*	This lab
5	*Phytophthora infestans*	This lab
6	*Sclerotium rolfsii Sacc.*	This lab
7	*Botrytis cinerea*	CGMCC
8	HP-24	This lab
9	BP-12	This lab
10	P10-7	Isolate

### 2.2 Isolation of antagonistic bacteria

Firstly, soil (5 g) was added to a 250 mL flask containing 45 mL sterile distilled water (SDW) and incubated on a rotary shaker at 150 rpm and 30 °C for 1 h to prepare the stock suspension. Next, 1 mL of the supernatant was taken and added to a test tube containing 9 mL of sterile water. The mixture was mixed thoroughly to obtain a concentration of 10^−1^. Then, this concentration was used as the sample and the above steps were repeated five times to achieve the required dilution for plate spreading (10^−6^). After that, 100 μL of the diluted solution was taken and spread evenly on an LB agar plate. The plate was incubated at 30 °C for 24 h. Finally, a single colony was picked, transferred, and purified in LB solid medium for characterization studies.

### 2.3 Screening of antagonistic bacteria against *Botrytis cinerea in vitro*

Screening was performed using the dual-culture plate assay with slight modifications (Zhou et al., 2022). A 5 mm-diameter mycelial plug of *B. cinerea* was placed in the center of a PDA plate. Ten different isolated and purified bacterial strains were inoculated at four equally spaced corners at a distance of 3 cm from the center. Plates were incubated at 25 °C for 7 days, and the formation of inhibition zones was observed and spore germination was observed under a light microscope (Heilman et al., 2013). The width of the clear inhibition zone was measured with vernier calipers. Each treatment was replicated three times. The mycelial growth inhibition rate (IR) was calculated using Ezrari’s method(Ezrari et al., 2021): IR = [(C - T) / C] × 100%, where C and T represent the average colony diameter of the fungus in the control and treatment groups, respectively.

### 2.4 Preparation of *Bacillus amyloliquefaciens* P10-7 fermentation supernatant and its effects on mycelium growth of *Botrytis cinerea*

Strain P10-7 was inoculated onto a culture LB plate. A loopful of the bacterial culture was transferred into a 50 mL sterile test tube containing 10 mL of LB culture medium. The tube was incubated overnight at 30 °C and 180 rpm (16–24 h). The concentration of the resulting bacterial suspension was adjusted to 1.0 × 10^8^ CFU/mL using a hemocytometer. Approximately 5 mL of this inoculum (1.0 × 10^8^ CFU/mL) was added to a 500 mL flask containing 250 mL LB broth and incubated at 180 rpm at 30 °C for 2 days. The culture broth was centrifuged at 4 °C and 8,000 × g for 20 min. The supernatant was collected and passed through a 0.22 μm filter membrane three times to obtain the *B. amyloliquefaciens* fermentation supernatant (BAFS) (Zhang et al., 2020).

The supernatant was collected and filtered three times through a 0.22 μm filter membrane. The resulting BAFS was diluted with sterile water to different concentrations for the determination of its effect on mycelial growth. The specific dilution procedure was performed as follows: the initial concentration of BAFS was mixed with sterile water at a 1:1 ratio to obtain a 2-fold diluted sterile filtrate. Subsequently, the 2-fold diluted solution was mixed with sterile water at a 1:1 ratio to obtain a 4-fold diluted sterile filtrate. In the way, 8-fold and 16-fold diluted sterile filtrates were obtained. The sterilized solid PDA medium was placed in a constant-temperature drying oven or water bath at 40 °C (to maintain liquidity) for cooling. The BAFS P10-7 dilutions of different concentrations were mixed with the unsolidified PDA solid medium at a 1:10 ratio (10 mL of filtrate added to 90 mL of PDA medium) and poured into 90 mm diameter petri dishes for subsequent experiments. PDA medium without BAFS was used as the control (CK). A 5 mm diameter plug of *B. cinerea* mycelium was placed in the center of the PDA medium and incubated at 25 °C until the control reached full plate size. The width of the zone of inhibition was measured using vernier calipers. Each treatment was repeated 3 times. The inhibition rate (IR) of the filtrate against the pathogen was calculated using the Ezrari method of section 2.3 (Ezrari et al., 2021).

### 2.5 Effect of strain P10-7 on spore germination of *Botrytis cinerea*

The effect of different concentrations of BAFS on *B. cinerea* morphology was observed using a dual-culture method (Cozzolino et al., 2020). Briefly, freshly cultured *B. cinerea* spores were eluted with sterile 0.1% tween solution and diluted to a concentration of 1.0 × 10^6^ spores/mL. BAFS (5 mL) was mixed well with 5 mL of the spore suspension. The mixture was incubated at 25 °C for 8 h. Using sterile tween solution as a control, 40 μL of the mixture was placed on a concave slide, and spore germination was observed under a light microscope (Heilman et al., 2013). Each treatment was replicated three times. The spore germination rate was calculated as: (number of germinated spores / total number of spores) × 100%. The spore germination inhibition rate (GIR) was calculated as: GIR = [(germination in control - germination in treatment) / germination in control] × 100%.

### 2.6 Effect of strain P10-7 on the mycelial growth of different plant pathogenic fungi

Characterization of the inhibitory effects of six pathogenic fungi using plate confrontation and BAFS assays was performed according to Ezrari (Ezrari et al., 2021). For the dual-culture assay, mycelial plugs (5 mm in diameter) of pathogenic fungi were placed in the center of PDA plates, and inoculated at 3 cm intervals at four equally spaced corners. For the BAFS assay, 1.5 mL of BAFS was mixed with 15 mL of liquefied PDA (cooled to 40 °C) to make a drug-containing plate, and the plate was placed with a cake of pathogenic fungi in the center. All plates were incubated at 28 °C for 7 days for the determination of bacterial inhibition.

### 2.7 Volatile organic compound strain P10-7

The inhibitory effect of Volatile organic compounds (VOCs) produced by P10-7 on *B. cinerea* was assessed using the sealed-plate method (Myo et al., 2019) with slight modifications. A 5-mm mycelial plug of actively growing *B. cinerea* was placed in the center of a PDA plate (top plate; inverted). This plate was inverted onto an LB plate inoculated with P10-7 culture. An LB plate inoculated with sterile broth served as the control. The two plates were sealed together, ensuring *B. cinerea* was exposed to VOCs released by P10-7. Three replicates per treatment were incubated at 25 °C for 5 d to observe the antagonistic effect.

### 2.8 Biochemical and metabolic enzymes of strain P10-7

Physiological and biochemical characterization was performed, including starch hydrolysis, catalase test (Lee et al., 2006), nitrate reduction, gelatin liquefaction, methyl red reaction, Voges-Proskauer (VP) test (Ji et al., 2014), and other standard tests (Xiu-Zhu and Miao-Ying, 2001).

Enzyme activities and metabolite production were determined using slightly modified methods (Demange et al., 1990; Syed-Ab-Rahman et al., 2018). P10-7 was inoculated into specific media for amylase, pectinase, protease, siderophore and *β*-1,3-glucanase production and incubated at 30 °C for 3 days. Amylase and pectinase media were stained with iodine solution for 5 min. *β*-1,3-glucanase medium was stained with 0.01% Congo Red for 15 min. Protease and siderophore media were observed directly. All treatments were performed in triplicate.

### 2.9 Stability of BAFS

BAFS was exposed to different temperatures, pH conditions and UV irradiation durations to determine stability, modifying the method of Li (Li et al., 2020).

#### 2.9.1 Temperature stability

BAFS P10-7 was treated at water bath conditions of 40 °C, 60 °C, 80 °C and 100 °C, as well as at high temperature (121 °C) for 20 min. After cooling to room temperature, the antibacterial activity of the filtrate against *B. cinerea* was determined according to section 2.5 LB liquid medium treated at the corresponding temperatures was used as a control instead of the fermentation filtrate, with each treatment repeated three times.

#### 2.9.2 pH stability

The sterile fermentation filtrate of the antagonistic bacterium P10-7 was adjusted to pH values of 3, 4, 5, 6, 7, 8, 9, 10 and 11 using 1 mol/L HCl and 1 mol/L NaOH, respectively. After incubating at 4 °C overnight, the filtrate was filtered through a 0.22 μm microporous membrane. The antibacterial activity of the original filtrate against *B. cinerea* was determined using the method described in section 2.5. LB liquid medium at the corresponding pH was used as a control in place of the fermentation filtrate, with each treatment repeated three times.

#### 2.9.3 UV stability

The sterile fermentation filtrate of the antagonistic strain P10-7 was placed under ultraviolet light irradiation. The irradiation conditions were set as follows: wavelength 254 nm, power 36 W, height 25 cm, with irradiation durations of 1 h, 2 h, 4 h, 8 h and 16 h. After irradiation, the antibacterial activity of the fermentation filtrate against *B. cinerea* was determined using the method described in section 2.5. LB liquid medium irradiated with the UV lamp for corresponding durations was used as a control, and each treatment was repeated three times.

### 2.10 Dynamics of strain P10-7 colonization in soil and on leaves

The colonization dynamics in soil and on leaves were studied with slight modifications to Yuan’s method (Yuan et al., 2022). Strain P10-7 cell suspension (1.0 × 10^7^ CFU/mL) was sprayed onto the rhizosphere soil and tomato leaves, respectively. Soil and leaf tissue samples (0.1 g) were collected at different time points.

The soil was added to 10 mL of sterile water and thoroughly mixed. Subsequently, the mixture was serially diluted with LB medium and spread onto agar plates. The leaves were placed into 2 mL centrifuge tubes and homogenized with 1 mL of sterile water. The homogenate was briefly centrifuged (2000 × g, 30 s) to obtain the supernatant, which was then subjected to serial dilution and plated onto LB medium. Colony counts were recorded after 24 h incubation at 30 °C. The experiment was performed in triplicate.

### 2.11 Effects of the biocontrol strain P10-7 on tomato seed germination and seedling growth

#### 2.11.1 Seed germination

Uniformly sized tomato seeds were surface-sterilized with 2% (v/v) sodium hypochlorite for 15 min, followed by 75% ethanol for 30 s, and rinsed three times with sterile water (Aiello et al., 2019). The experiment containing five treatment methods: four different concentrations of P10-7 fermentation broth and sterile water (CK control group). The fermentation broth was prepared according to method 2.4 and diluted with water to different concentrations. Sterilized tomato seeds were soaked for 4 h in different concentrations of P10-7 biocontrol bacterial suspension (1.0 × 10^8^ CFU/mL, 1.0 × 10^7^ CFU/mL, 1.0 × 10^6^ CFU/mL, 1.0 × 10^5^ CFU/mL) or sterile water (control). Seeds were then transferred to Petri dishes containing double-layer filter paper moistened with 3 mL sterile water. All dishes were incubated at 28 °C. Radicle length was measured after 4 days (Mussa et al., 2018).

#### 2.11.2 Seedling growth

One hundred tomato plants with consistent growth conditions were selected and divided into 5 treatment groups. The cell suspension P10-7 was diluted with water to concentrations of 1.0 × 10^8^ CFU/mL, 1.0 × 10^7^ CFU/mL, 1.0 × 10^6^ CFU/mL and 1.0 × 10^5^ CFU/mL. Each treatment group received 10 mL of cell supernatant for root irrigation of the plant seedlings. The treatment was repeated thrice at 7 days interval. The control group received sterile water. Data were collected on the 21st day after the first irrigation treatment. The tomato plants were rinsed with water, surface moisture was absorbed with absorbent paper, and assessments were made of plant height, root length, fresh weight, and dry weight (Chen et al., 2021).

### 2.12 Effect of strain P10-7 on tomato gray mold under greenhouse conditions

Disinfected tomato seeds were sown in 12 × 6-well trays(Wang et al., 2020). After 2 weeks, the seedlings were transplanted into nutrient pots, with 30 tomato seedlings per treatment sample. *B. cinerea* was cultured on PDA solid medium using a 0.1% Tween 80 wash, gently shaken for 30 min to collect the spore suspension, and adjusted to a concentration of 1.0 × 10^7^ spores/mL using a hemocytometer for later use. The preparation of the biocontrol fungus spore suspension followed method 2.4, with water dilutions to concentrations of 1.0 × 10^5^ CFU/mL, 1.0 × 10^6^ CFU/mL, 1.0 × 10^7^ CFU/mL and 1.0 × 10^8^ CFU/mL. When tomato seedlings develop 4–5 true leaves, the inoculum was applied via leaf spraying. They were cultivated at 25 °C, 80% relative humidity for 2 days. The prepared biocontrol bacterial fermentation liquid was sprayed onto the plants until it no longer dripped. The control group received water treatment.

The incidence, disease index, and control effect on the tomato plants were assessed on the 20th day after biocontrol treatment. The disease severity index of *B. cinerea* on tomato plants was defined as the percentage of leaf area in a diseased state, where 0 indicates no disease symptoms, 1 indicates 0.1–25%, 2 indicates 25.1–50%, 3 indicates 50.1–75% and 4 indicates 75.1–100% (Wang et al., 2018). The disease severity value for each plant was calculated using the following formula: Disease rate = number of diseased leaves/total number of leaves surveyed × 100%, Disease Index = {(∑ [number of diseased leaves × disease severity index])/ (total number of leaves surveyed × highest disease severity level)} × 100. The formula for calculating Control Efficiency is as follows: Disease control = ([A − B]/A) × 100%, where A is the disease severity caused solely by pathogen inoculation, and B is the disease severity after various treatments.

### 2.13 Genome sequencing and annotation

P10-7 genomic DNA was extracted from 0.1 g of bacterial tissues using the optimized SDS extraction method, and P10-7 whole genome sequencing and assembly were done by Tianjin Extreme Intelligence Biotechnology Co., Ltd. using Nanopore PromethION and Illumina NovaSeq 6,000 platforms. The raw results of sequencing were processed and assembled using Unicycler (version: 0.50) (Wick et al., 2017) and Pilon v2.0. The predicted coding sequences were annotated from Pfam, NR, COG, SwissProt, GO, and KEGG databases using sequence alignment tools like Diamond, BLAST, and HMMER. Biosynthetic gene clusters (BGCs) were predicted using antiSMASH software. The P10-7 whole genome sequencing data have been accessed at NCBI under accession number CP182393.

### 2.14 Phylogenomic analysis

The obtained sequences were compared with genomes in the GenBank database of the National Center for Biotechnology Information using BLAST-N software.^1^ Genome sequences of various *Bacillus* species were downloaded, and genome trees were constructed through genome comparison using the online tools M1CR0B1AL1Z3R^2^ and iTOL.^3^ Genome-wide comparisons of P10-7 and its homologous sequences were conducted using the Mauve Multi-Genome Comparison Tool. Average Nucleotide Identity (ANI) tools^4^ were employed to estimate the similarity of the genome sequences of the different *Bacillus* species studied.

## 3 Results

### 3.1 Isolation, purification, and screening of antagonistic strains

**Figure 1:**
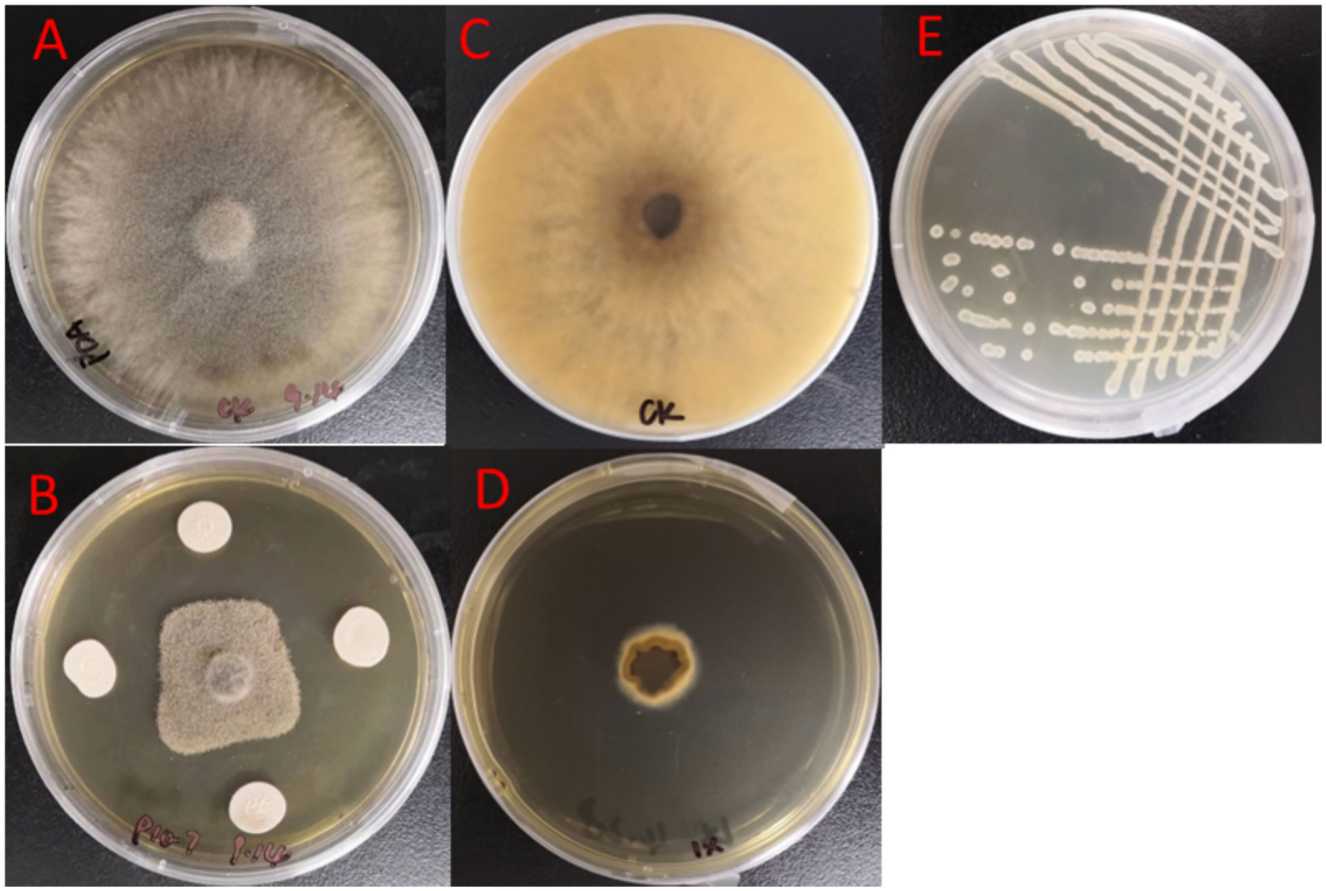
Identification of bacterium P10-7 and its antagonistic activity against *B. cinerea* under different conditions. **(A,C)** Pathogenic bacteria cultured only on PDA medium **(B)** Dual culture of P10-7 against pathogen on PDA; **(D)** Pathogen on PDA amended with fermentation broth of P10-7; **(E)** Colony morphology of P10-7 on LB solid medium.

From the 40 rhizosphere soil samples, a total of 118. Initial screening against *B. cinerea* using the dual-culture assay identified 10 strains exhibiting antagonism, with inhibition rates ranging from 48.35 to 74.77% (Table 2). Among these, strain P10-7 demonstrated the strongest inhibitory effect (74.77% inhibition rate, Table 2; Figure 1B). PDA agar plates were completely colonized by the strain *B. cinerea* (25°C, 7 days), with the plate surfaces and undersides were shown in Figures 1A,C. Colonies of P10-7 on LB agar (30 °C, 24 h) were white, moist, round, and exhibited a wrinkled surface morphology (Figure 1E).

**Table 2:** Effect of selected bacterial strains on the mycelial growth of *B. cinerea in vitro.*

Strain number	Inhibition rate /%
P1-2	60.66 ± 1.38bc
P2-3	56.16 ± 3.75 cd
P4-12	52.85 ± 3.16d
P6-4	48.65 ± 1.56d
P8-9	63.96 ± 3.60b
P8-12	55.26 ± 2.27 cd
P10-6	57.66 ± 3.25c
P10-7	74.77 ± 1.80a
HP-24	63.06 ± 2.38b
BP-12	48.35 ± 2.08d

The inhibition rates (%) (*n* = 3, mean ± SE). Different letters within each table indicate significant differences between treatments (*p* < 0.05).

The results of BAFS concentrate treatment with antagonistic bacteria were shown in Figure 1D. Notably, BAFS exhibited a significantly higher inhibition rate (92.09%) than the P10-7 cell suspension in the dual-culture assay (74.77%). Moreover, the inhibitory effect of BAFS decreased progressively with dilution (from 92.09 to 21.79%; Supplementary Table S1). Based on the superior inhibitory activity of the sterile filtrate (BAFS), subsequent experiments focused on investigating its effects on *B. cinerea* mycelial growth and spore germination.

### 3.2 Effect of strain P10-7 on *Botrytis cinerea* mycelium growth and spore germination

**Figure 2:**
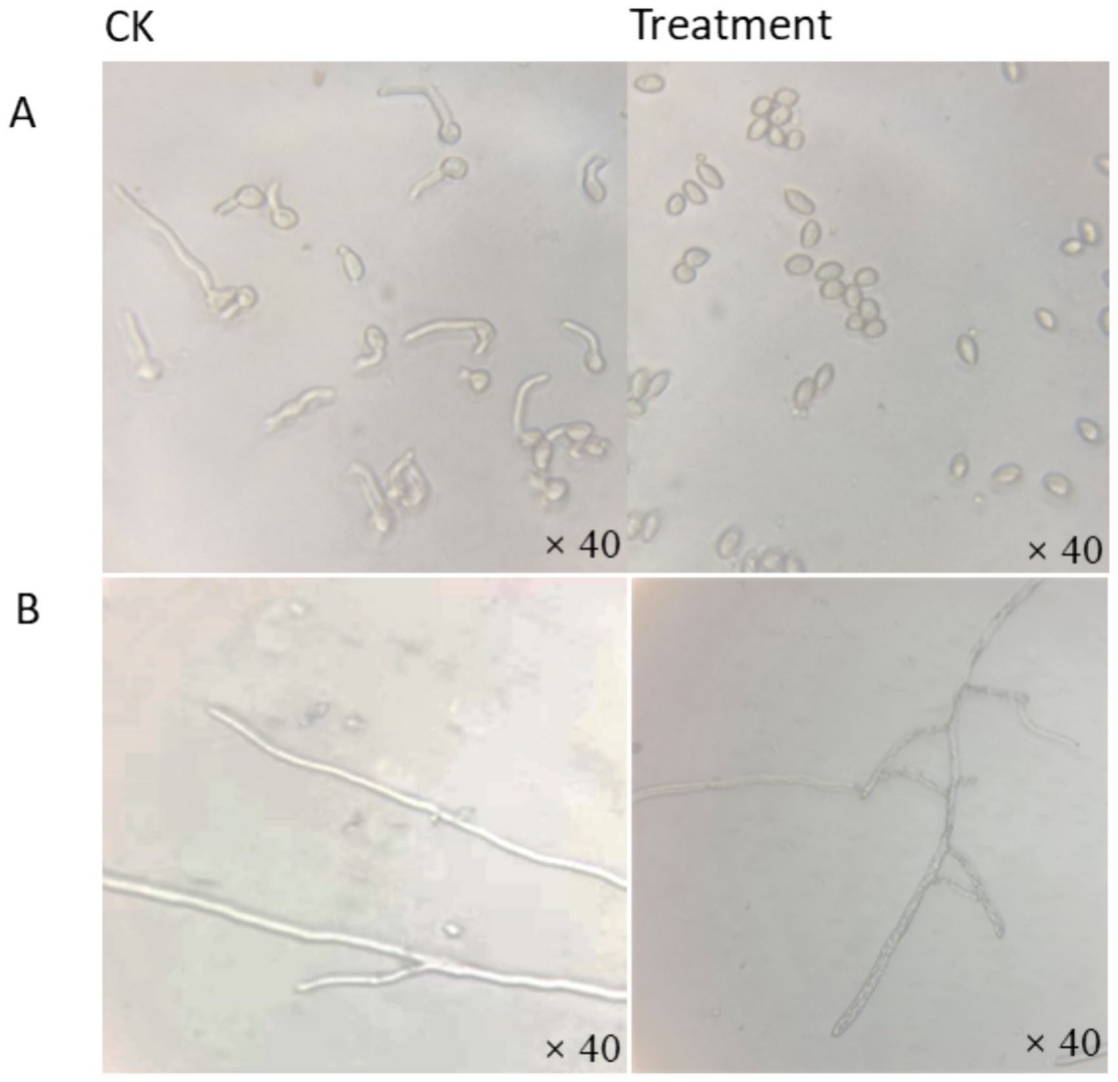
Microscopic observation of mycelial growth and spore germination of *B. cinerea* after treatment with the strain P10-7 **(A)** spore germination. **(B)** Microscopic observation of mycelium treatment.

BAFS profoundly inhibited *B. cinerea* spore germination (Figure 2A). After 8 h incubation at 25 °C, the germination rate in the control (CK) was 91.83%, whereas in the BAFS treatment group it was only 1.80%, resulting in a germination inhibition rate (GIR) of 98.03%. In addition to suppressing spore germination, BAFS also induced severe morphological alterations in *B. cinerea* hyphae (Figure 2B). Microscopic observation revealed that the hyphae in untreated control plate had a smooth surface, uniform thickness and clear spacing (Figure 2B). P10-7 treated hyphae showed uneven thickness, bending, breakage, cytoplasmic coagulation and leakage (Figure 2B). The results showed that P10-7 was able to inhibit both the mycelial growth and spores germination of *B. cinerea*.

### 3.3 Broad-spectrum antagonism of P10-7

**Figure 3:**
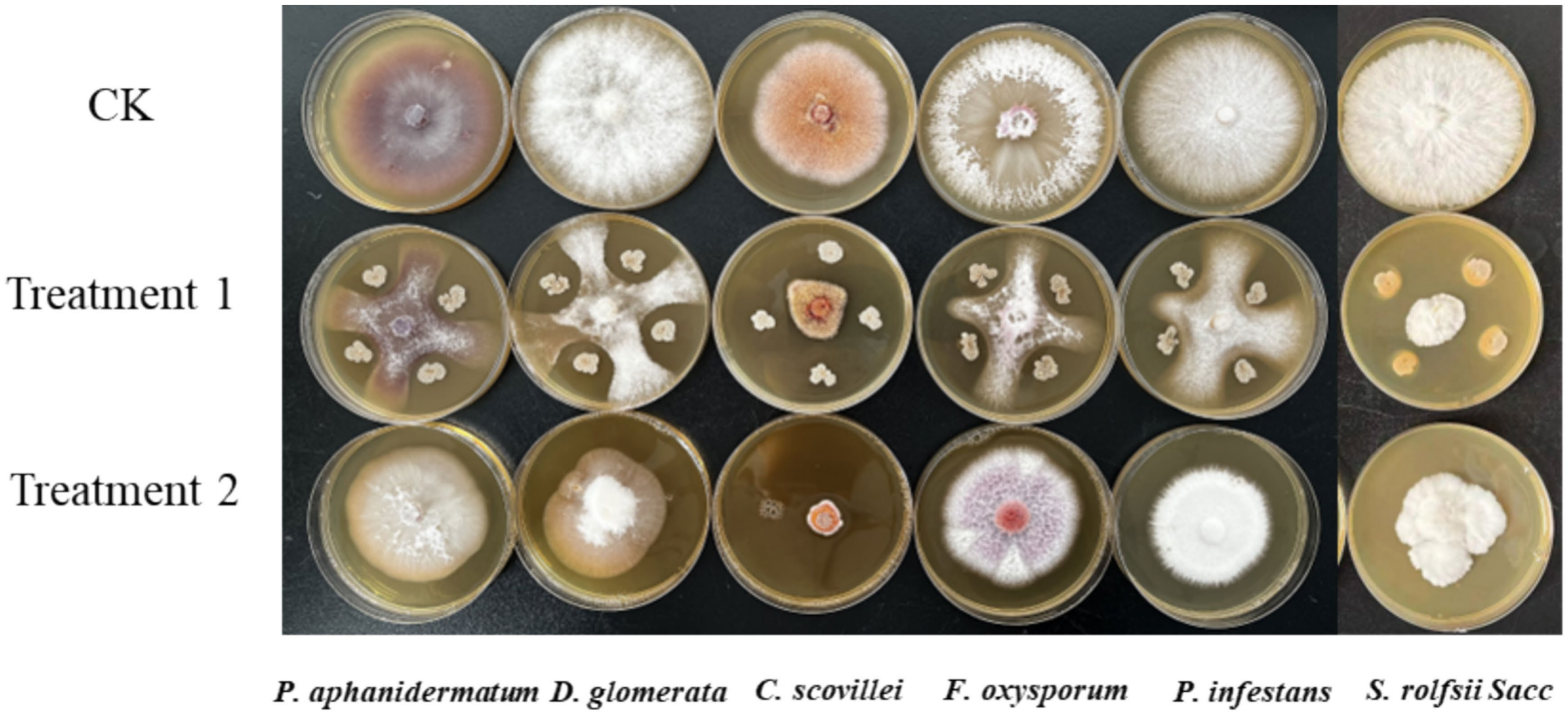
Inhibitory effects of P10-7 against fungal phytopathogens CK: only pathogen on PDA; Treatment 1: dual culture of P10-7 against pathogen on PDA; Treatment 2: pathogen on PDA amended with fermentation broth of P10-7.

P10-7 exhibited varying degrees of antagonism against six plant pathogens. The inhibition rates of the cell suspension and BAFS against the pathogens ranged from 55.30–72.51% and 21.05–87.51%, respectively (Figure 3; Table 3). The pattern of inhibition varied with the pathogen and the method used. For example, BAFS had an inhibition rate of 87.51% against *Colletotrichum scovillei*, which was higher than the 70.14% inhibition rate of the cell suspension. This result was consistent with the findings in Section 3.1, where BAFS showed a higher inhibition effect against *B. cinerea* than cell suspension. Conversely, for the majority of other pathogens tested, the inhibition effect of the cell suspension on the rest of the pathogens was much higher than that of BAFS. This broad-spectrum activity highlights the significant biocontrol potential of strain P10-7.

**Table 3:** Effect of strain P10-7 on the mycelial growth of different fungal plant pathogens.

Strains number	Name of the pathogens	Inhibition rate /%	
		Cell suspension	BAFS
1	*Pythium aphanidermatum*	63.41 ± 1.15c	21.05 ± 2.71d
2	*Didymella glomerata*	72.51 ± 2.62a	44.16 ± 2.25b
3	*Colletotrichum scovillei*	70.14 ± 0.53ab	87.51 ± 3.69a
4	*Fusarium oxysporum*	67.43 ± 1.68b	23.25 ± 7.22 cd
5	*Phytophthora infestans*	56.04 ± 2.21d	27.05 ± 3.99c
6	*Sclerotium rolfsii Sacc.*	55.30 ± 3.28d	28.03 ± 9.93c

The inhibition rates (%) (*n* = 3, mean ± SE). Different letters within each table indicate significant differences between treatments (*p* < 0.05).

### 3.4 Detection of antagonism-related lytic enzymes and metabolites

**Figure 4:**
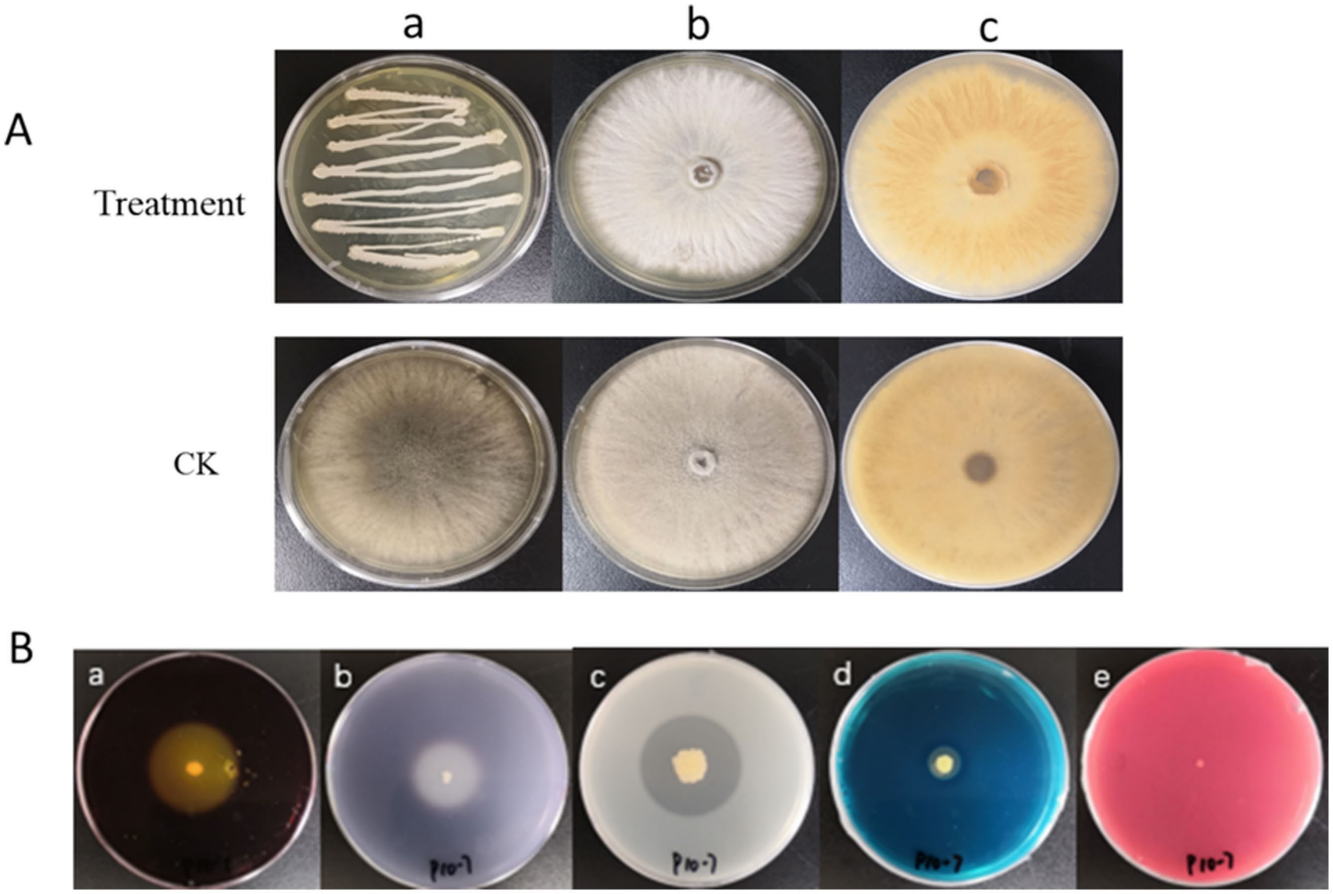
Antagonistic activity of strain P10-7 against *B. cinerea* volatiles and determination of P10-7 enzyme activity **(A)** Antagonistic detection of *B. cinerea* and P10-7 VOCs; **(A-a)** Growth conditions of the lower dish cover; **(A-b)** Growth conditions on the front of the upper plate cover; **(A-c)** Growth conditions on the back of the upper plate cover; **(B)** Detection of extracellular enzyme production of P10-7, **(B-a)** pectinase; **(B-b)** amylase; **(B-c)** protease; **(B-d)** siderophore; **(B-e)** *β*-1,3-glucanase.

Many Volatile organic compounds VOCs produced by *Bacillus*, such as alcohols, acids, aldehydes, ketones and esters, inhibited phytopathogenic fungi (El Jaddaoui et al., 2023). As seen in Figure 4A *B. cinerea* grew in both control and treated plates. Assessment of VOCs produced by P10-7 revealed a clear impact on *B. cinerea* morphology (Figure 4A). Although growth occurred in both control and VOC-exposed plates, the mycelium exposed to P10-7 VOCs appeared lighter in color and, observed from the plate bottom, was tightly adhered to the medium and exhibited significant wrinkling (Figures 4A-b,c), suggesting impaired growth.

Physiological and biochemical test results are shown in Supplementary Table S2, More importantly, results for enzyme activities and secondary metabolites (Figure 4B) showed that strain P10-7 produced pectinase, amylase, protease, and siderophores, but did not produce *β*-1,3-glucanase. This profile of secondary metabolites contributes to its high biocontrol potential.

### 3.5 Stability of BAFS

**Figure 5:**
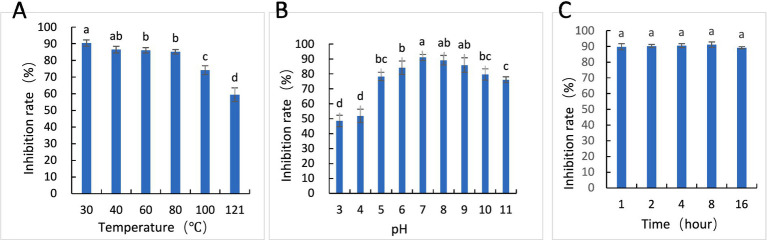
Stability of *B. amyloliquefaciens* P10-7 fermentation supernatant (BAFS). Stability of BAFS at **(A)** different temperatures, **(B)** different pH values, and **(C)** different UV irradiation times. Bars indicate standard error (± SE). *p* < 0.05 was considered significantly different from each group.

Experimental results showed that the antifungal activity of BAFS decreased when heated above 80 °C but exhibited good thermal stability overall (Figure 5A). The lowest inhibition rate was observed at pH 3 and 4, while the strongest inhibition (92.09%) occurred at pH 7. BAFS remained stable in weakly acidic, neutral, and alkaline environments, showing wide pH tolerance (Figure 5B). Under UV irradiation for up to 16 h, the inhibition rate remained above 90%, indicating strong stability (Figure 5C). In summary, BAFS of P10-7 has a high degree of stability.

### 3.6 Analysis of colonization dynamics of strain P10-7 in soil and on leaves

**Figure 6:**
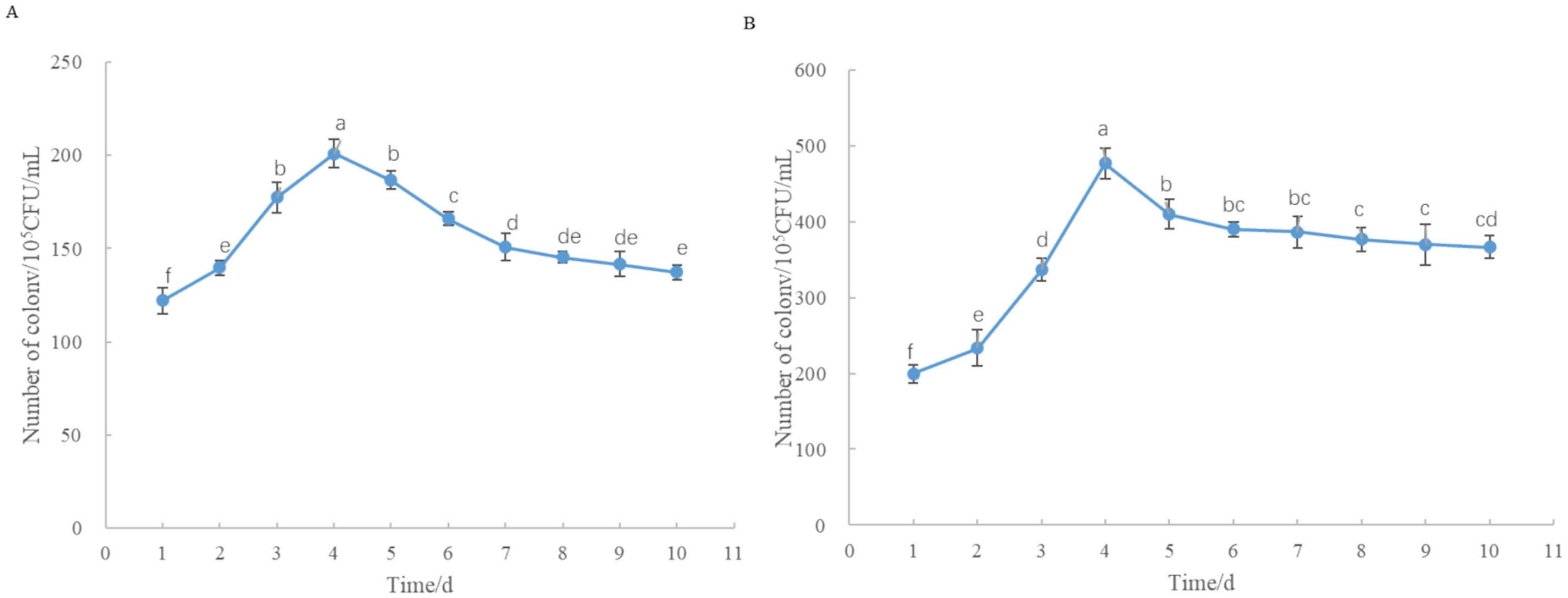
Colonization dynamics of strain P10-7 in soil and leaves. **(A)** Soil. **(B)** leaf of tomato. Data are presented as mean ± standard error (*n* = 3). Vertical bars indicate standard deviation from the mean. Analysis of variance revealed significant differences between treatment groups. Different letters within each figure indicate significant differences between treatments (*p* < 0.05).

Statistical results (Figure 6) showed that after spraying on soil and leaves, P10-7 populations increased rapidly, both of which peaked on the 4th day at 2.0 × 10^7^ CFU/mL and 4.7 × 10^7^ CFU/mL, respectively, where the number of colonies in the leaves was greater than that in the soil at the same time, probably due to the influence of other microorganisms received from the soil. The number of colonies in the soil and the leaves tended to stabilize on the 10th day, which was 1.4 × 10^7^ CFU/mL and 3.7 × 10^7^ CFU/mL, respectively.

### 3.7 Effects of strain P10-7 suspension on tomato seed germination and seedling growth

The effects of different P10-7 concentrations on tomato seeds were investigated (Supplementary Table S3). At a spore concentration of 1.0 × 10^7^ CFU/mL, seed germination rate increased by 6% and root length increased 1.16-fold compared to the control, representing the optimal concentration for seed germination. At a bacterial suspension concentration of 1.0 × 10^7^ CFU/mL, P10-7 significantly promoted tomato seedling growth: maximum plant height and shoot length were 1.3 times higher than the CK (sterile water) group, root length was 1.54 times higher, fresh weight was 1.7 times higher, and dry weight was 1.5 times higher. The highest growth-promoting effect was observed at 1.0 × 10^7^ CFU/mL (Table 4).

**Table 4:** Effect of P10-7 strain on seed germination and seedling vigor of tomato.

Indicators	CK	P10-7			
		1.0 × 10^8^	1.0 × 10^7^	1.0 × 10^6^	1.0 × 10^5^
Height/cm	21.40 ± 2.63c	24.57 ± 1.60b	27.97 ± 0.42a	23.00 ± 0.40bc	22.57 ± 0.60bc
Root Length/cm	9.87 ± 1.01b	14.50 ± 1.05a	15.27 ± 2.84a	13.43 ± 2.32ab	11.90 ± 2.69ab
Fresh weight/g	5.99 ± 0.17c	6.79 ± 0.96c	10.37 ± 0.83a	7.54 ± 0.13b	7.40 ± 0.25b
Dry Weight/g	0.40 ± 0.01c	0.43 ± 0.06c	0.63 ± 0.05a	0.48 ± 0.02b	0.47 ± 0.03bc

The inhibition rates (%) (*n* = 3, mean ± SE). Different letters within each figure indicate significant differences between treatments (*p* < 0.05).

### 3.8 Biocontrol effect of P10-7 against tomato gray mold

Pot experiments showed that symptoms appeared on single leaves 5 days after inoculation with *B. cinerea*. By day 20, the disease index in the control group reached 30.57%. Disease incidence and disease index in all P10-7 treatment groups were lower than in the control (CK). The optimal concentration was 1.0 × 10^7^ CFU/mLin which disease incidence and disease index were only one-sixth of the control, and the control efficacy reached 80.35% (Table 5), indicating high effectiveness.

**Table 5:** Effect of different concentrations of P10-7 on tomato gray mold.

Treatment	Disease rate/%	Disease index	Control efficiency%
CK	48.01 ± 1.74a	30.57 ± 2.03a	
1.0 × 10^8^	13.27 ± 1.51d	7.94 ± 1.56 cd	74.02 ± 5.10ab
1.0 × 10^7^	8.51 ± 1.29e	6.01 ± 1.27d	80.35 ± 4.15a
1.0 × 10^6^	18.20 ± 1.71c	9.76 ± 0.83c	68.08 ± 2.70b
1.0 × 10^5^	24.54 ± 2.39b	14.40 ± 1.97b	52.90 ± 6.45c

The inhibition rates (%) (*n* = 3, mean ± SE). Different letters within each table indicate significant differences between treatments (*p* < 0.05).

### 3.9 Genome sequencing of the P10-7 genome

**Figure 7:**
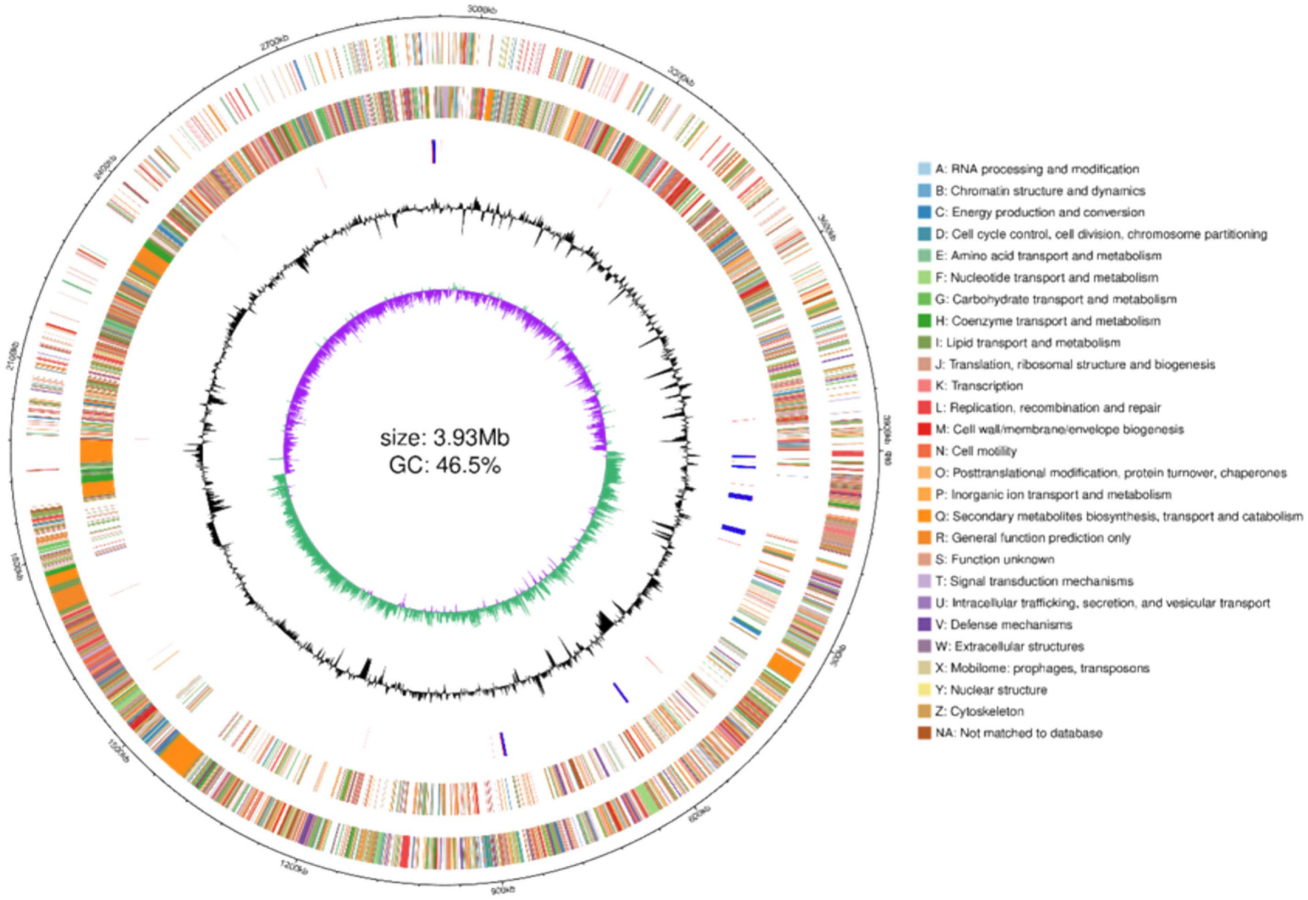
Genome map and annotation of strain P10-7. Genome map of strain P10-7. The circles from 1 to 6 (outer to inner) represent markers of genome size, CDS on the forward strand, CDS on the reverse strand, rRNA and tRNA, GC content and GC-skew.

To elucidate the genomic characteristics of P10-7 and predict its potential biopreventive mechanisms, the entire genome of P10-7 was sequenced using a combination of three-generation Nanopore sequencing and two-generation illumina sequencing. The genome was circularly mapped with circlize software. The total length of P10-7’s genome was 3,929,792 bp, with an average GC content of 46.5%, comprising 3,747 coding sequences (CDSs) and 86 tRNA genes (Figure 7).

### 3.10 Analysis and characterization of sequencing data of the P10-7 genome

**Figure 8:**
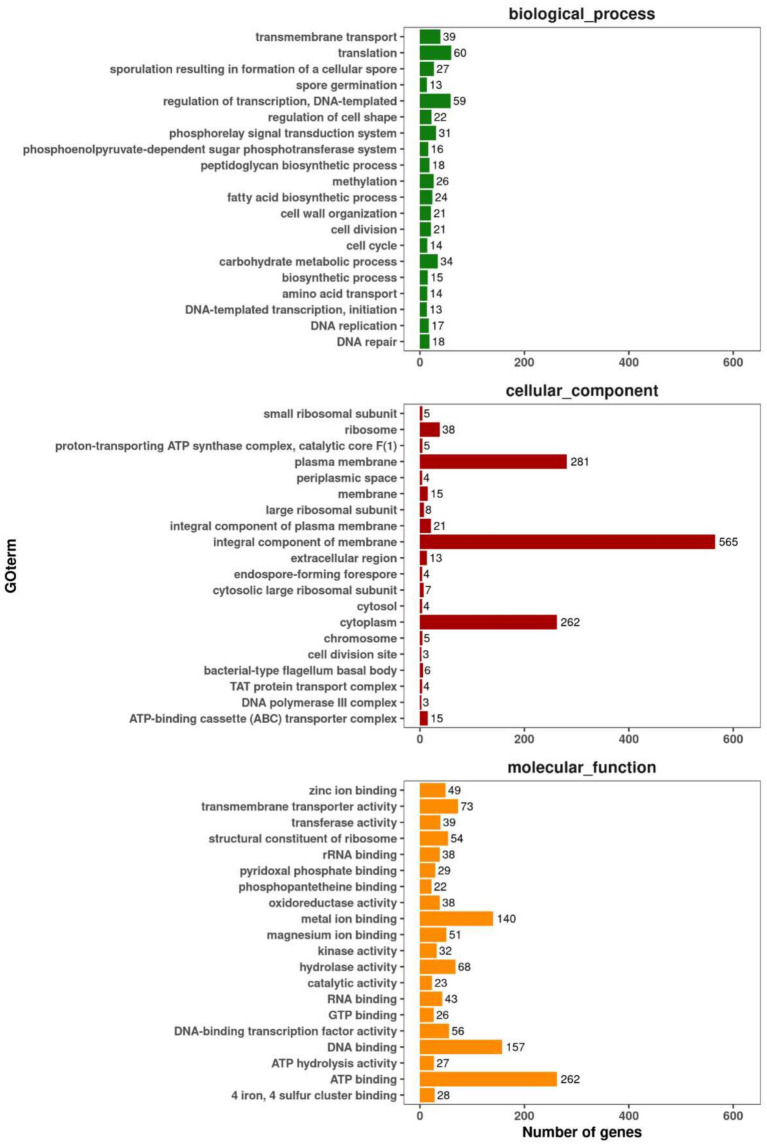
Gene ontology (GO) annotation and functional classification of P10-7.

The percentage of total coding genes annotated to the GO database and categorized into biological processes, cellular components and molecular functions was 56.42% (2,114 genes) (Figure 8).

**Figure 9:**
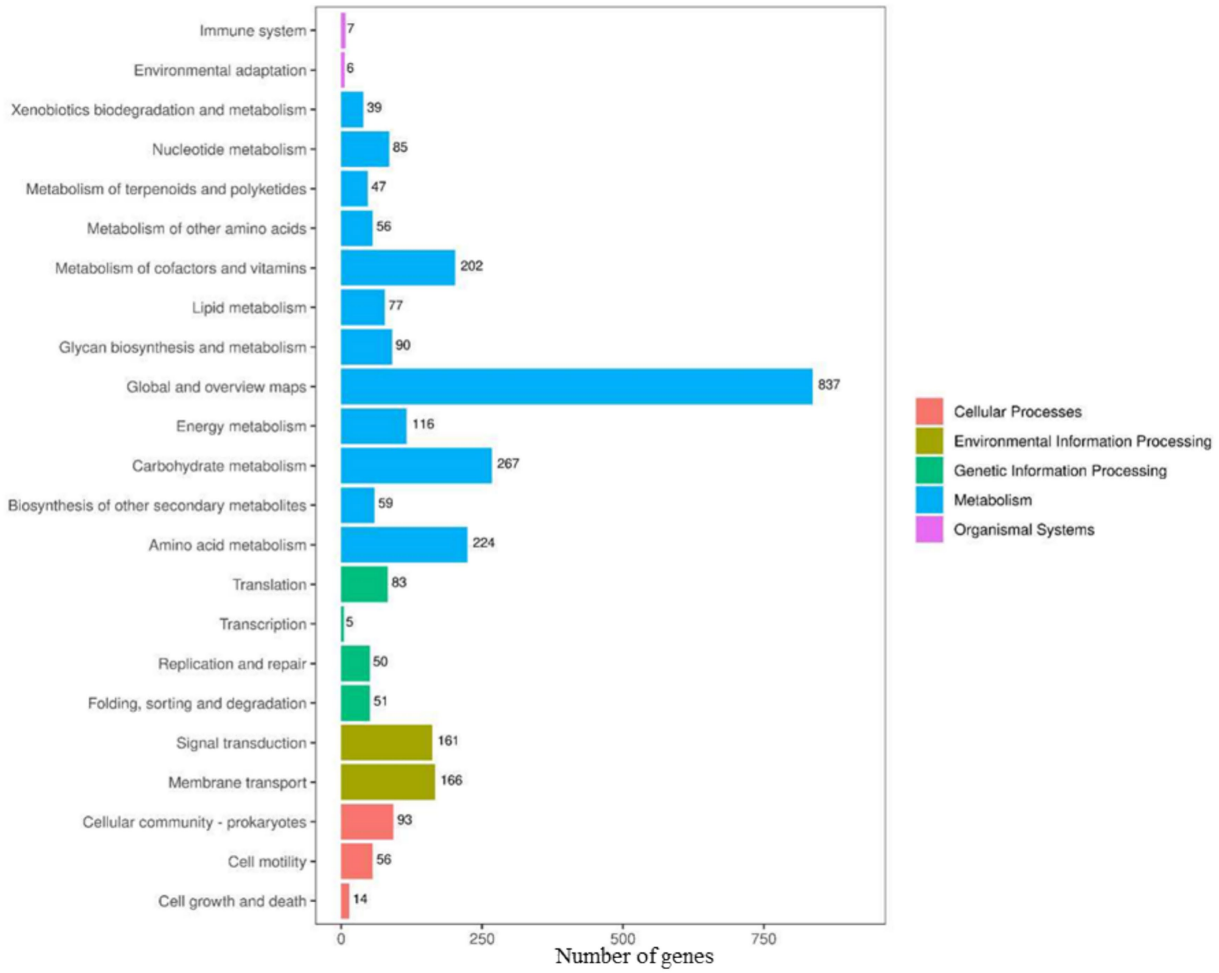
Functional classification of P10-7.

**Figure 10:**
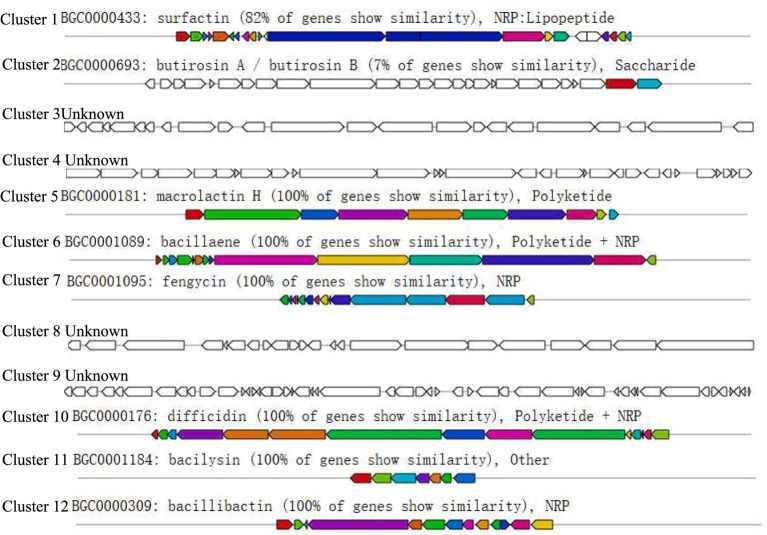
Genome map and annotation of strain P10-7. Secondary metabolite synthesis gene clusters.

There were 502 genes in the biological process category, most of them involved in transcriptional regulation; of these 1,263 genes, most of them were related to membrane components, especially cell membrane and cytoplasmic functions, and belong to the cytogenetic category; and most of the genes in the molecular function category were categorized as ATP-binding, DNA-binding and metal ion-binding. In addition, for the 2,791 genes (66.16%) in the KEGG annotation, the top three categories were global and overview map (837), carbohydrate metabolism (267), and amino acid metabolism (224) (Figure 9). The antiSMASH software predicted 12 biosynthetic gene clusters (BGCs) in the P10-7 genome sequence (Figure 10). These BGCs were classified into seven groups: four NRPS and transATPKS, T3PKS, betalactone, PKS-like, RiPP-like, and other types. Among these, six BGCs showed high similarity to the known BGCs related to macrolactin H (100%), bacillaene (100%), fengycin (100%), difficidin (100%), bacillibactin (100%), bacilysin (100%), butirosin A / butirosin B (7) and surfactin (82%). Notably, some BGCs showed very low similarity or were unknown, implying that P10-7 probably produces new metabolites.

### 3.11 P10-7 shares significant similarity with *Bacillus*

**Figure 11:**
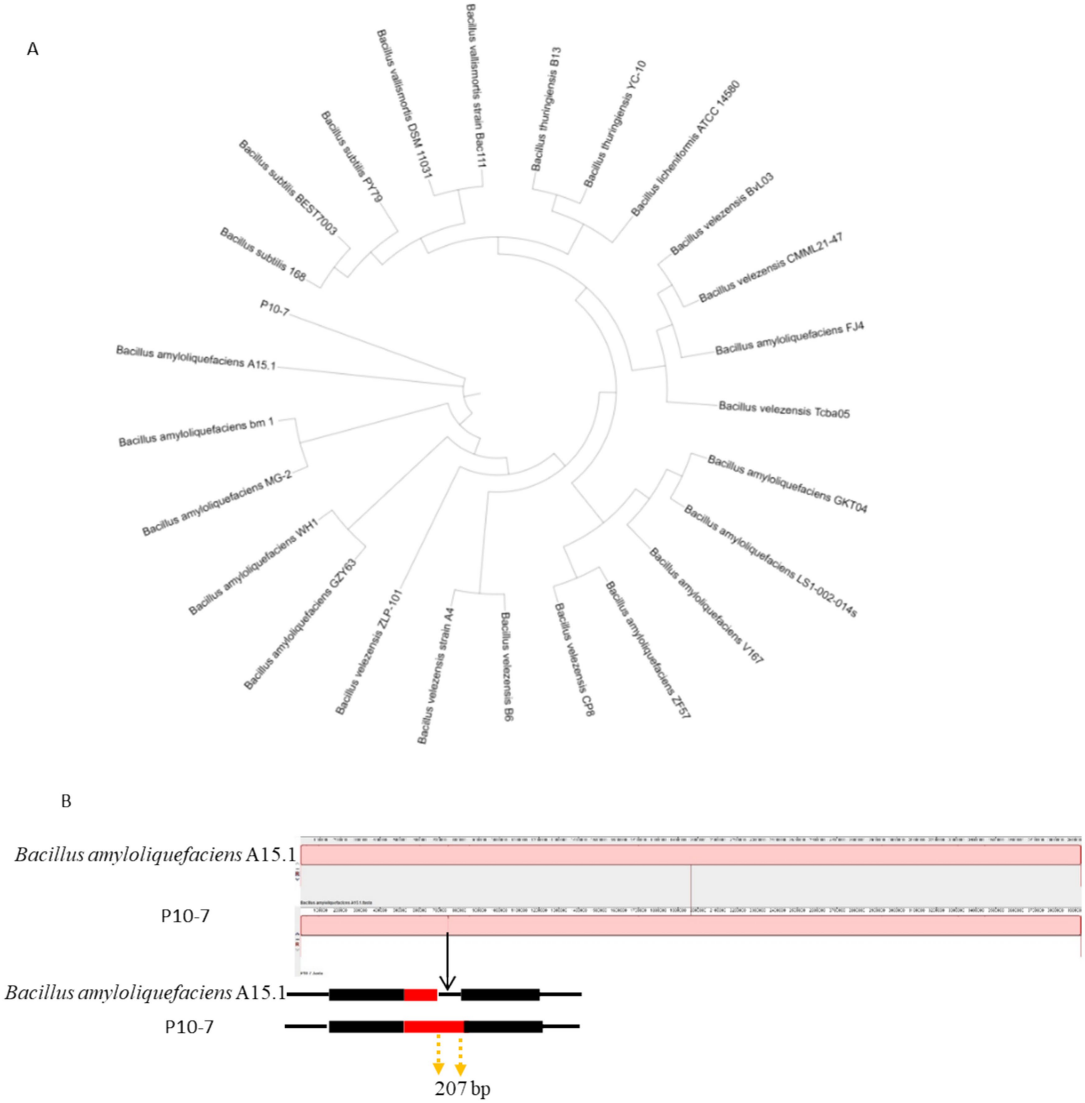
Genome-wide comparison. **(A)** Phylogenomic relationship between the P10-7 strain and various species of the *Bacillus* genus; **(B)** Multiplex comparison of the whole genome sequences of strains P10-7 and *B. amyloliquefaciens* A15.1 using Mauve (version 2.4.0).

**Figure 12:**
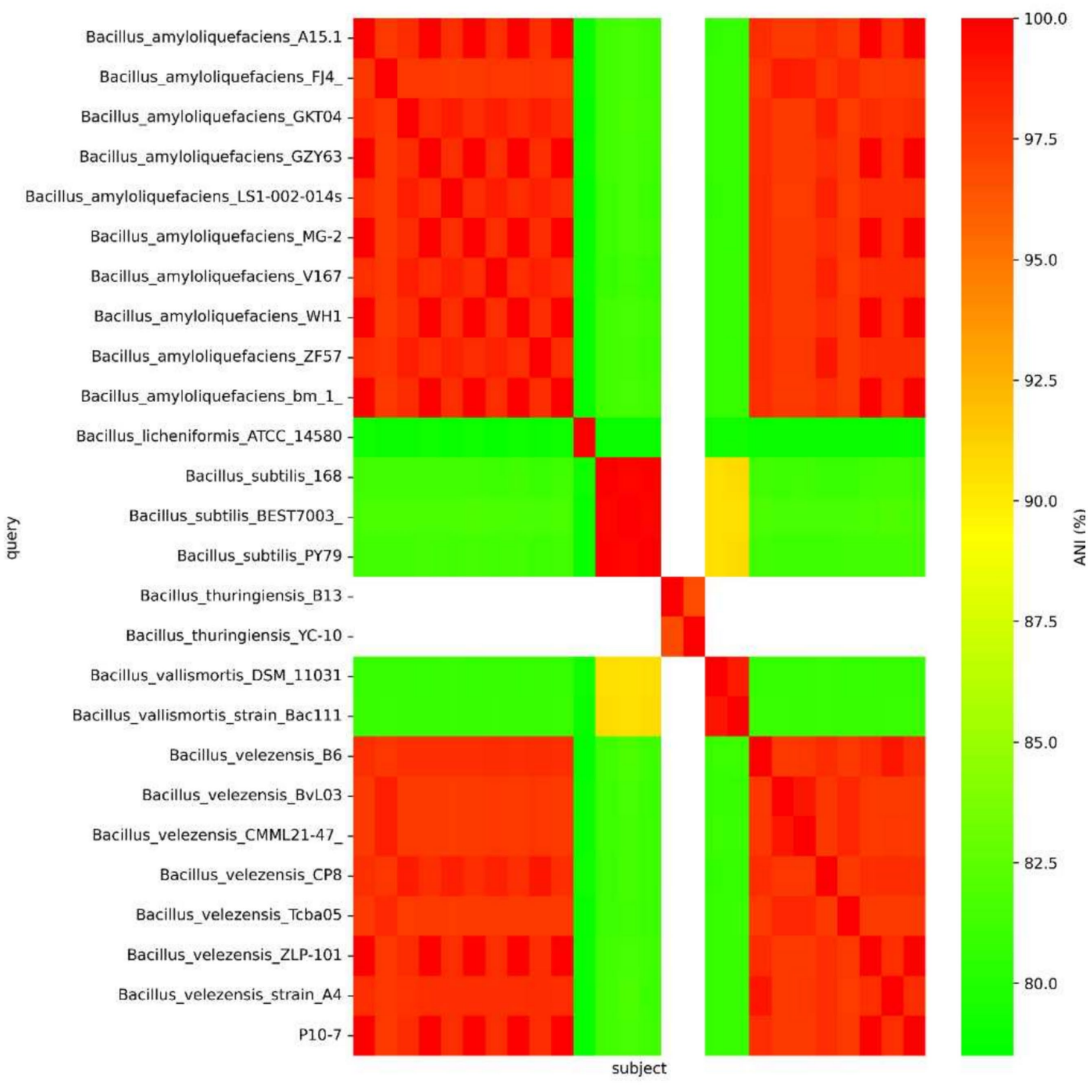
Heatmap of average nucleotide identity (ANI) values for whole genomes of the strain *Bacillus* P10-7 and 19 other *Bacillus* species.

The complete genome sequences of 25 *Bacillus* species, including *B. virescens*, *B. amyloliquefaciens*, *B. licheniformis*, *B. subtilis*, *B. vallismortis*, *B. velezensis* and *B. thuringiensis*, were selected through BLAST-N analysis. The constructed phylogenetic tree indicated that the strain was closely related to *B. amyloliquefaciens* (Figure 11A). Whole-genome comparisons between P10-7 and *B. amyloliquefaciens* A15.1 were conducted using the Mauve Multi-Genome Comparison Tool. The sequence alignment revealed that P10-7 contained an additional 207 bp DNA repeat fragment, potentially resulting from the mutational evolution of the *B. amyloliquefaciens* A15.1 strain (Figure 11B). Furthermore, genome sequence similarity analysis demonstrated that P10-7 shared a high average nucleotide identity (ANI) of 99–100% with both *B. subtilis* and *B. amyloliquefaciens* (Figure 12).

## 4 Discussion

In this study, we isolated a bacterial strain (P10-7) exhibiting broad-spectrum antagonism from tomato rhizosphere soil. Several *Bacillus* species with antifungal properties, such as *B. maritimus* B-9987 (Gu et al., 2017) and *B. velezensis* SL-6 (Cozzolino et al., 2020), have been isolated previously, confirming the suitability of *Bacillus* spp. for biocontrol. Due to the limitations of 16 S rRNA gene sequencing for differentiating closely related *Bacillus.* strains (Wang et al., 2023), a polyphasic approach combining morphological, physiological, biochemical, and whole-genome sequencing analyses was employed. This led to the accurate identification of strain P10-7 as *B. amyloliquefaciens.* We further performed an ANI sequence comparison between strain P10-7 and other Velezensis sequences, which resulted in the results as shown in Figure 11A, with *B. velezensis* and *B. amyloliquefaciens* having the highest ANI values (Figure 12), which exceeded the species-defining threshold (Richter and Rosselló-Móra, 2009), again proving that strain P10-7 is *B. amyloliquefaciens*. Phylogenomic analysis further revealed high homology between P10-7 and *B. amyloliquefaciens* strain A15.1, with the primary difference being a 207-bp repetitive sequence insertion at position 742,610 bp in P10-7 (Figure 11B), suggesting it may represent an evolutionary variant. Although both strains originate from China, their geographical isolation (>3,000 km apart) and the lack of prior functional characterization of A15.1 highlight the novelty of P10-7.

*Bacillus siamensis*, *B. velezensis*, and *B. amyloliquefaciens* are *Bacillus* species reported to be effective against various plant pathogens, including *B. cinerea*, with *B. velezensis* receiving significant attention (Tian et al., 2023). For instance, *B. velezensis* QSE-21 showed strong antagonism (72.23% inhibition on PDA) (Yang et al., 2021), L33a showed 75.22% inhibition *in vitro* (Sun et al., 2024), and P10-7 isolated here showed a similarly high inhibition rate of 74.77% (Table 2).

Hyphae represent the primary invasive form of plant pathogenic fungi during invasion and colonization. Many studies have demonstrated that biocontrol agents inhibit pathogens by suppressing spore production and mycelial growth and altering hyphal morphology (Feng et al., 2021; Dengbo et al., 2022). Accordingly, *in vitro* assays with different concentrations of BAFS P10-7 demonstrated inhibition of *B. cinerea* through suppression of spore germination and alteration of hyphal morphology. Similarly, Li (Li et al., 2020) found that the filtrate of *B. amyloliquefaciens* BA 17 inhibited *B. cinerea* by reducing conidial germination and mycelial growth. The biocontrol effect of BAFS decreased with dilution (Figure 2; Supplementary Table S1).

The antagonistic activity of many biocontrol microorganisms, including *Bacillus*, is often mediated by antimicrobial secondary metabolites. Li et al. (2022), such as surfactin and bacillins, which are effective in controlling pathogens by disrupting cell membranes or limiting nutrient sources (Wang et al., 2023). In this study, antiSMASH analysis revealed that the P10-7 genome contains 12 BGCs, which were predicted to biosynthesize some compounds, including fengycin, surfactin, bacillaene, butirosin A / butirosin B, bacillibactin, macrolactin H, difficidin, bacilysin and some unknown compounds (Figure 10). Notably, these unknown compounds imply that P10-7 may produce new secondary metabolites, whose functional mechanisms need to be further explored using knockout techniques. New studies have shown that VOCs produced by some biocontrol microorganisms have excellent biocontrol properties (Chaouachi et al., 2021; Srikamwang et al., 2023). Similarly, VOCs produced by P10-7 exhibited inhibitory activity against *B. cinerea* (Figure 4A), although the specific compounds involved require further identification and characterization.

Beyond secondary metabolites, the production of lytic enzymes and siderophores plays a significant role in biocontrol. Siderophore production, for instance, is an initial mechanism (Kloepper et al., 1980). *Bacillus* produces various secondary metabolites inhibitory to pathogens, including proteases that directly inhibit pathogen growth (Martínez-Absalón et al., 2014), pectinases that may stimulate plant resistance (Bodhankar et al., 2017; Bhagwat et al., 2019), and siderophores that sequester environmental iron, limiting pathogen growth (Martínez-Absalón et al., 2014). Consistent with its strong *in vitro* efficacy against *B. cinerea* (92.09% mycelial inhibition, 98.03% spore germination inhibition by BAFS), strain P10-7 was confirmed to produce key antagonistic compounds including protease, amylase, pectinase, and siderophores (Figure 4B). These metabolites are likely central to its observed antifungal activity. While *β*-1,3-glucanase can decompose fungal cell walls and stimulate plant resistance, strain P10-7 did not produce this enzyme (Figure 4B).

Bacterial communities primarily inhabit and interact within the rhizosphere and phyllosphere (leaf surface). Both are complex ecosystems where bacteria play crucial roles, including protecting plants from pathogens (Raklami et al., 2019; Fadiji et al., 2022). The colonization ability of biocontrol bacteria is closely linked to their efficacy and stability (Kloepper and Beauchamp, 1992). Our study demonstrated that P10-7 although was isolated from soil, but it showed good colonization ability on soil and leaves (Figure 6), maintaining high population densities for 10 days post-treatment. BAFS also exhibited high stability under varying temperature, pH, and UV conditions (Figure 5), demonstrating strong application potential.

This study confirmed the positive effect of strain P10-7 on tomato seed germination and seedling growth. These results, consistent with its biocontrol efficacy, highlight the strain’s significant potential. The growth-promoting and disease-controlling effects of P10-7 could benefit tomato production. Biocontrol of destructive plant pathogens aligns with the demand for green and healthy agricultural products driven by low residue levels. In the greenhouse pot experiment, P10-7 achieved 80.35% control efficacy against tomato gray mold at 1.0 × 10^7^ CFU/mL. These results provide a theoretical basis for the preventive effect of strain P10-7 in field conditions. Typically, effective biocontrol agents inhibit pathogens both *in vitro* and *in vivo* (Lining et al., 2021; Dengbo et al., 2022). This study demonstrated that BAFS significantly reduced the incidence and severity of gray mold in tomato (Table 5), confirming the biocontrol capability of P10-7 metabolites.

## 5 Conclusion

In conclusion, a novel antifungal strain, identified as *B. amyloliquefaciens* P10-7, was isolated from tomato rhizosphere soil. This strain exhibits broad-spectrum antagonism against seven plant pathogenic fungi in both dual-culture and BAFS assays. Importantly, P10-7 demonstrated high efficacy in controlling *B. cinerea* under greenhouse conditions, significantly reducing disease incidence. Furthermore, P10-7 application promoted tomato seed germination and seedling growth. Genomic analysis revealed the presence of 12 biosynthetic gene clusters (BGCs), including those responsible for known antifungal metabolites and potentially novel compounds, alongside genes encoding key lytic enzymes and siderophores. The high stability of its bioactive metabolites (BAFS) under varying environmental conditions (temperature, pH, UV), combined with its effective colonization ability in soil and on leaves, further supports the biocontrol potential of P10-7. Collectively, *Bacillus amyloliquefaciens* P10-7 represents a highly promising candidate for the development of an effective and environmentally friendly biocontrol agent against tomato gray mold.

## Data Availability

The datasets presented in this study can be found in online repositories. The names of the repository/repositories and accession number(s) can be found in the article/Supplementary material.

## Footnotes

^1^ https://blast.ncbi.nlm.nih.gov/Blast.cgi

^2^ https://microbializer.tau.ac.il/

^3^ https://itol.embl.de/

^4^ http://enve-omics.ce.gatech.edu/ani/
