# Supplementary material for: Isolation and characterization of new antagonistic bacteria P10-7 and evaluation of its biocontrol potential against tomato gray mold
Source: Front Microbiol. 2025 Sep 18;16:1668865. doi: 10.3389/fmicb.2025.1668865 (PMC12488616; doi:10.3389/fmicb.2025.1668865)
Supplement: Supplementary file 1 [file Table_1.DOCX]

**Table S 1.** Inhibition of Staphylococcus griseus by BAFS.

| Dilution factor | Inhibition rate % |
| --- | --- |
| 1 | 92.09±1.61a |
| 2 | 73.08±3.39b |
| 4 | 63.46±2.94c |
| 8 | 36.54±4.84d |
| 16 | 21.79±3.39e |
