# Supplementary material for: Isolation and characterization of new antagonistic bacteria P10-7 and evaluation of its biocontrol potential against tomato gray mold
Source: Front Microbiol. 2025 Sep 18;16:1668865. doi: 10.3389/fmicb.2025.1668865 (PMC12488616; doi:10.3389/fmicb.2025.1668865)
Supplement: Supplementary file 2 [file Table_2.DOCX]

**Table S 2.** Physiological and biochemical characteristics of P10.

Symbol “+” indicates positive and “–” indicates negative.

| Characteristics | Strain P10-7 |
| --- | --- |
| contact enzyme reaction | + |
| Oxidase activity reaction | + |
| Liquefaction of gelatin | + |
| Starch hydrolysis reaction | + |
| Methyl red test | - |
| VP test | + |
| Citrate utilization | + |
| Propionate utilization | - |
| Nitrate reduced to nitrite | + |
| D-xylose acid production | + |
| L-arabinose acid production | + |
| D-mannitol acid production | + |
