# Supplementary material for: Isolation and characterization of new antagonistic bacteria P10-7 and evaluation of its biocontrol potential against tomato gray mold
Source: Front Microbiol. 2025 Sep 18;16:1668865. doi: 10.3389/fmicb.2025.1668865 (PMC12488616; doi:10.3389/fmicb.2025.1668865)
Supplement: Supplementary file 3 [file Table_3.DOCX]

**Table S 3.** P10-7 Promoter effects of strains on tomato seeds.

| Treament | Germination rate | Germination rate /% | Root length/cm |
| --- | --- | --- | --- |
| CK | 27.67±0.58b | 79.05±1.65b | 8.29±0.72a |
| 10^8^ | 27.00±1.73b | 77.14±4.95b | 7.86±1.00a |
| 10^7^ | 30.00±1.00a | 85.71±2.86a | 9.63±0.63a |
| 10^6^ | 28.67±1.53ab | 81.90±4.36ab | 8.42±1.94a |
| 10^5^ | 28.00±1.00ab | 80.00±2.86ab | 8.38±1.55a |

The inhibition rates (%) (n = 3, mean ± SE). Different letters indicate significantly different groups (p < 0.05).
